# Use of nasal mucosa graft in tympanoplasty

**DOI:** 10.1016/j.bjorl.2020.06.006

**Published:** 2020-07-21

**Authors:** Sandro Barros Coelho, Willian da Silva Lopes, Gabriela de Andrade Meireles Bezerra, Davi Farias de Araújo, Adriano Sérgio Freire Meira, Sílvio da Silva Caldas Neto

**Affiliations:** a Universidade Federal do Ceará, Hospital Universitário Walter Cantídeo, Fortaleza, CE, Brazil; b Universidade Federal do Maranhão, Otoclínica, Imperatriz, MA, Brazil; c SOS Otorrino Clínica de Otorrinolaringologia, João Pessoa, PB, Brazil; d Universidade Federal de Pernambuco, Recife, PE, Brazil

**Keywords:** Tympanoplasty, Nasal mucosa, Clinical trial, Endoscopic surgical procedure

## Abstract

**Introduction:**

Tympanoplasty techniques with different types of graft have been used to close tympanic perforations since the 19th century. Tragal cartilage and temporalis fascia are the most frequently used types of graft. They lead to similar functional and morphological results in most cases. Although little published evidence is present, nasal mucosa has also been shown to be a good alternative graft.

**Objective:**

Surgical and audiological outcomes at the six-month follow-up in type I tympanoplasty using nasal mucosa and temporalis fascia grafts were analyzed.

**Methods:**

A total of 40 candidates for type I tympanoplasty were randomly selected and divided into the nasal mucosa and temporalis fascia graft groups with 20 in each group. The assessed parameters included surgical success; the rate of complete closure of tympanic perforation and hearing results; the difference between post- and pre-operative mean quadritonal airway-bone gap, six months after surgery.

**Results:**

Complete closure of the tympanic perforation was achieved in 17 of 20 patients in both groups. The mean quadritonal airway-bone gap closures were11.9 and 11.1 dB for the nasal mucosa and temporalis fascia groups, respectively. There was no statistically significant difference between the groups.

**Conclusion:**

The nasal mucosa graft can be considered similar to the temporal fascia when considering the surgical success rate of graft acceptance and ultimate audiological gain.

## Introduction

Tympanoplasty using a skin graft^1^ was first achieved with surgical success by Berthold in 1878. Since then, several otolaryngologists and researchers have further developed this procedure. In the early 20th century, new technologies and equipment, such as surgical microscopes, antibiotics, and general anesthesia created a favorable scenario for conducting tympanoplasty surgery using a skin graft. In 1956, Wüllstein classified tympanoplasty in five types.^2^ Some years later, Shea started to use veins as a graft with an underlay technique.^3^ Storrs and Patterson introduced the temporalis fascia graft (TFG),^4^ which produced favorable results among the classical surgeries.^5^ Underlay tympanoplasty using fascia temporalis graft became the worldwide gold standard. Many case series were published demonstrating its good surgical and audiological results, with a low level of complications.^6^, ^7^, ^8^ Since then, different graft materials with a focus on tragal cartilage have been used as alternative to TFG.^9^ In the last decade, many systematic reviews and meta-analyses have been published in which similar functional and morphological results between cartilage and the TFG have been shown excluding large perforations, reoperations or auditory tube malfunction, and cases in which cartilage grafts were demonstrated to be superior.^10^, ^11^, ^12^, ^13^, ^14^, ^15^

The use of the nasal mucosa as a graft (NMG) was developed as an alternative to the tragus and TFG grafts. One of its main positive aspects is the histological similarity with middle ear mucosa as shown in some recent studies. Hamma and colleagues developed a cell sheet derived from nasal cells to create an artificial middle ear mucosal that was designed for postoperative cholesteatoma treatment.^16^ Yamamoto and colleagues developed a method to transplant autologous nasal mucosal epithelial cell sheets to damaged middle ear cavities in an animal model. The results showed that a post-transplanted middle ear was morphologically and functionally similar to a normal middle ear.^17^, ^18^ Strasser and colleagues used autologous nasal mucosa as a transplant for covering tympanic membrane defects in 12 patients, yielding complete closure in 11 of them.^19^ The use of nasal mucosa as a graft, however, has been described in only a few studies. To our knowledge, no study comparing NMG to other grafts in tympanoplasty was published until the current one.

Efforts to find better grafts for tympanoplasty are still undergoing development in the scientific community. The objective of this study was to compare surgical and audiological results in type I tympanoplasty using NMG and TFG at the six-month follow-up.

## Methods

Participants who were candidates for type I tympanoplasty were selected among patients at a tertiary health center. The study was previously approved by the national research ethics committee (Protocol 2.397.367 CAAE: 50318215.8.0000.5045).

### Informed consent

All procedures performed in studies involving human participants were in accordance with the standards ethics of the institutional and/or national research committee and with the 1964 Helsinki declaration and its amendments or comparable ethical standards. Informed consent was obtained from all participants who were included in the study.

### Inclusion criteria

Patients with dry central (non-wet and non-marginal) tympanic perforation for at least 60 days, age between nine and 60 years old, absence of previous surgery in the studied ear, absence of retraction pockets or large perforation, and absence of active smoking or active nasal diseases were candidates for a graft and were included in the study. Inclusion criteria were designed to select the best candidates for TFG. Patients who had Eustachian tube dysfunction, large perforations, and previous unsuccessful tympanoplasties were not included in the group.

### Exclusion criteria

Exclusion criteria were loss of followup, presence of retraction pockets after surgery (to avoid bias of possible dysfunction of the auditory tube), and chronic diseases, such as *diabetes mellitus*, systemic arterial hypertension, and hypo- or hyperthyroidism. Patients with any clinical conditions that could interfere with the results of the surgery were also excluded.

### Surgical technique

**Figure 1:**
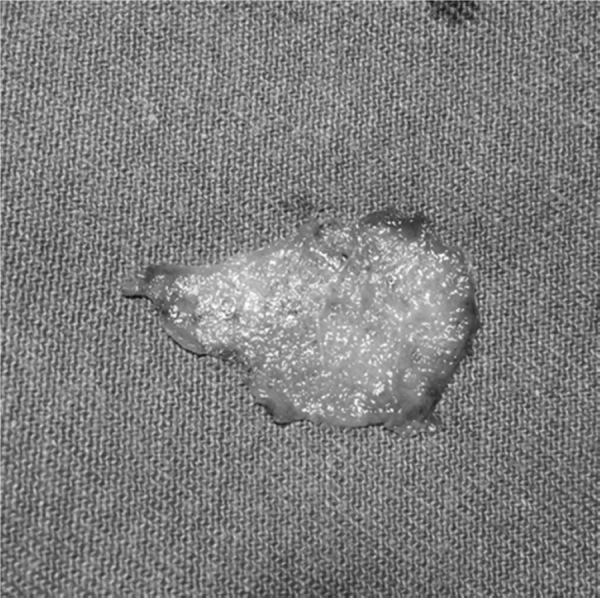
Nasal mucosa graft before preparation.

**Figure 2:**
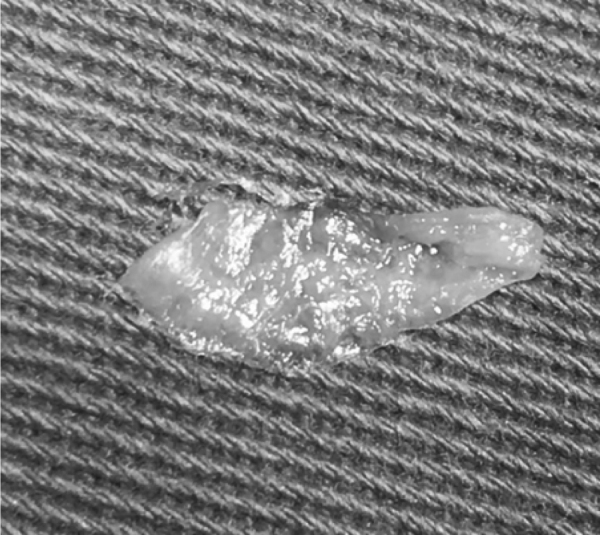
Nasal mucosa graft after preparation.

**Figure 3:**
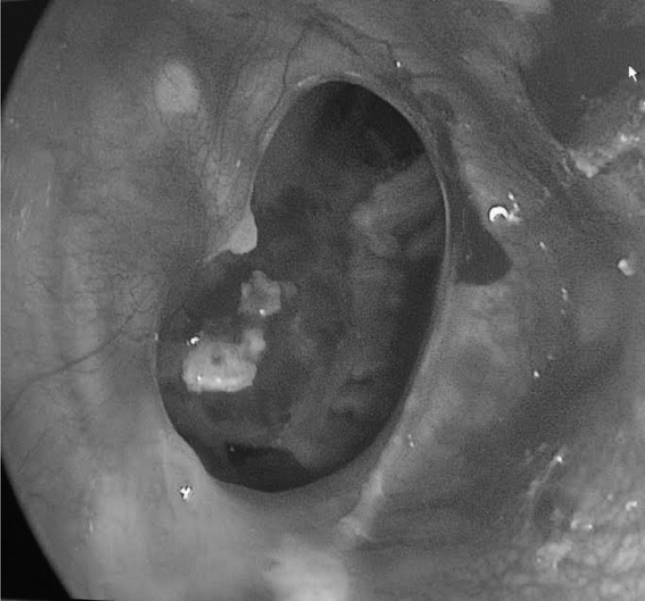
Preoperative aspect of tympanic perforation.

**Figure 4:**
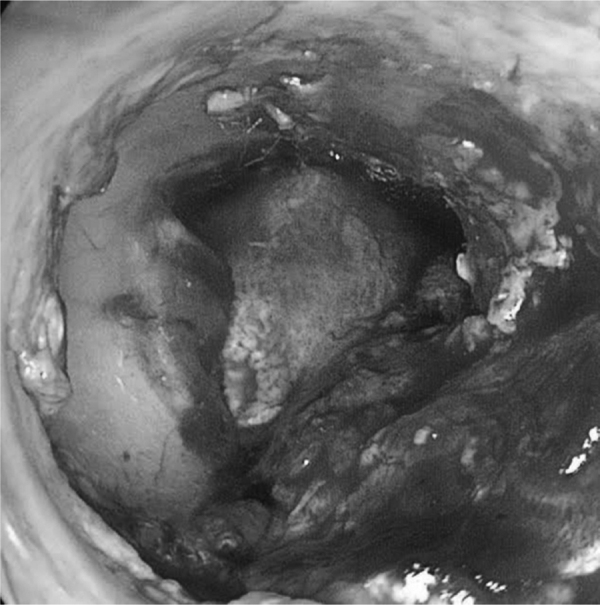
Nasal mucosa graft positioning during surgery.

Study participants were randomly assigned to different groups (NMG and TFG) by selecting names out of a container a few minutes prior to surgery. If any statistically significant difference between groups was observed after forming them, random adjustment would be performed. All patients underwent endoscopically-assisted type I tympanoplasty under general anesthesia using an underlay technique with Gelfoam™ for the graft and flap fixation after tympanic perforation border scarification. Temporalis fascia was harvested from the supra- auricular region and was used wet. In the TFG group, no nasal surgery was performed. In the NMG group, before nasal surgery, a thorough nasal cleaning with a saline solution for antisepsis was performed. The nasal mucosa was harvested in the contralateral inferior nasal turbinate head by using a small anterior turbinectomy of the size needed for the graft. Hemostasis was performed by electrocauterization and Gelfoam™. After collection, the NMG was separated from its submucosal tissue together with any piece of attached bone. The NMG appearance after its preparation was very similar to TFG features, except for a stickier consistency (Figure 1, Figure 2). After positioning in the patient’s ear, the submucosal aspect of NMG was always placed facing the external auditory canal to improve regeneration as the blood face will further stimulate the scar tissue reaction with the edges of the tympanic perforation and avoid adhesions with middle ear mucosa (Figure 3, Figure 4).

All patients underwent post-operative followups for six months. They attended the first followup medical appointment a week after surgery to remove stitches or remove scabs when necessary. No oral antibiotics were used in the postoperative period. Ear drops containing ciprofloxacin and dexamethasone were used in the second week for seven days. One month after surgery, a new examination was performed to examine the success of the surgery. Two months after surgery, audiological tests were performed to evaluate audiological gain. Six months after surgery, the last medical examination was carried out to again observe the surgical success and record any complications that may have occurred during this period.

### Surgical success

Partial closure or no closures of tympanic perforation were considered failures.

### Audiological gain

Audiological gain was measured by difference between post- and pre-operative mean quadritonal (500 Hz, 1000 Hz, 2000 Hz and 4000 Hz) airway-bone gap.

### Statistical analysis

The Kolomogorov-Smirnov test was applied to assess normality and to check the statistical distribution of the results. When parametric results were found, a Chi-Square test was used to analyze surgical success rate, while Student’s *t*-test was used to verify airway-bone gap closure. Non-parametric results were analyzed with the Kruskal-Wallis test. A 95% Confidence Interval (CI) was accepted for all statistical analysis.

## Results

### Patients

Forty-two patients were selected from January 2016 to October 2018. Two patients were excluded due to loss of followup. The remaining 40 patients were distributed at random into the TFG and NMG groups with 20 in each group. The mean age was 30.7 ± 14.3 years. The study included 27 female and 13 male patients. No statistically significant difference in age was observed between genders (Table 1).

**Table 1:** Demographic distribution among study groups.

Variable	TFG	NMG	Total	*p*-value
Female, n (%)	13 (65.0%)	14 (70.0%)	27 (67.5%)	0.731
Male, n (%)	7 (35.0%)	6 (30.0%)	13 (32.5%)	0.731
Age (mean ± SD)	26.9 ± 4.7	34.6 ± 7.9	30.7 ± 14.3	0.091

n, number of patients; SD, Standard Deviation; TFG, Temporalis Fascia Graft group; NMG, Nasal Mucosa Graft group.

### Surgical success

**Figure 5:**
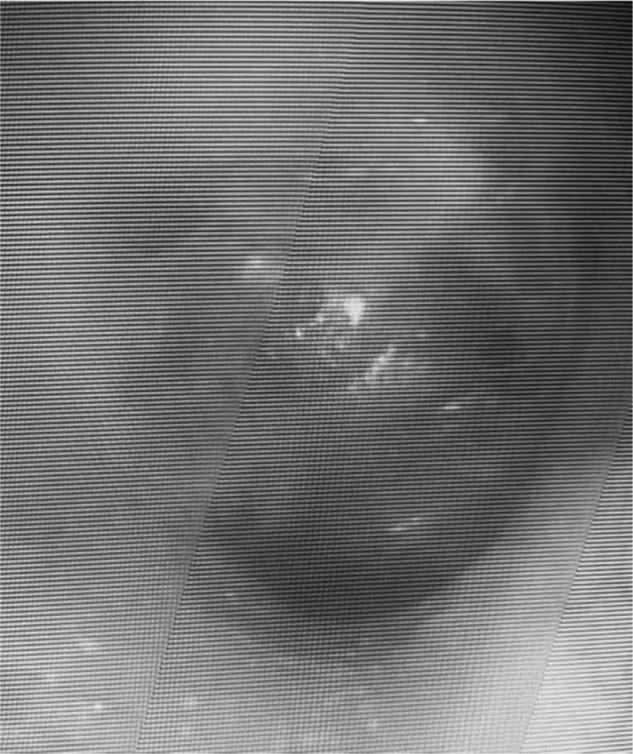
Postoperative aspect of nasal mucosa graft.

Both groups achieved 17 successful graft acceptances out of 20 total cases per group (Fig. 5). Partial or no closure of tympanic perforations were considered failures, which corresponded to three cases out of the 20 in each group. Importantly, otorrheas were not observed in these cases. The surgical success rate was 85% for both groups (Table 2).

**Table 2:** Comparison of surgical success between each group.

Variable	TFG	NMG	Total	*p*-value
Success, n (%)	17 (85.0%)	17 (85.0%)	34 (85.0%)	1.000
Fail, n (%)	3 (15.0%)	3 (15.0%)	6 (15.0%)	1.000

n, number of patients; TFG, Temporalis Fascia Graft group; NMG, Nasal Mucosa Graft group.

We did not observe difficulties during surgical procedures. NMG handling required a little more skill due to the stickier consistency of NMG that caused it to adhere to to surgical instruments, but this stickiness did not present any major technical problems during surgery.

Infection or other types of major complications were not observed. Epistaxis or the need for compressive dressing were not observed.

### Audiological gain

The NMG group achieved an average air-bone gap closure of 11.9 dB, whereas the TFG group showed a mean audiological gain of 11.1 dB. There was no statistical difference between groups concerning audiological parameters (Table 3).

**Table 3:** Mean quadritonal (500, 1000, 2000 and 4000 Hz) airway-bone gap by study group and mean’s comparison test result.

Variable	TFG	NMG	*p*-value	
			*t*-test	KW
Pre-operative (mean ± SD)	23.2 ± 3.6	23.6 ± 3.4	−	0.783
Post-operative (mean ± SD)	11.6 ± 4.1	11.7 ± 3.3	−	0.775
Airway-bone gap closure (mean ± SD)	11.1 ± 2.8	11.9 ± 3.9	0.727	−

SD, Standard Deviation; TFG, Temporalis Fascia Graft group; NMG, Nasal Mucosa Graft group; KW, Kruskal-Wallis test.

## Discussion

There is a consensus in the literature that both TFG and cartilage of tragus graft will present similar results with respect to audiological gain and rate of tympanic perforation closure.^19^, ^20^, ^21^, ^22^, ^23^ TFG was chosen as the control due to its previously described effectiveness and security in addition to its texture and consistency, which is closer to NMG. Wet TFG was selected due to better published results compared to dry TFG and shorter surgical time.^24^ In addition, the underlay technique was used as it presents better surgical outcomes when compared to overlay technique.^25^, ^26^

The surgical success rate was 85% for both groups (*p* = 1.000) after six months of followup examinations. Previous meta-analyses and systematic reviews regarding temporalis fascia showed similar rates of graft integration that ranged from 80% to 90%.^10^, ^11^, ^12^, ^13^, ^14^, ^15^, ^26^ This result suggests that NMG is as satisfactory as TFG with regard to rate of graft acceptance.

Regarding audiological gain, there was an average improvement of 11.5 dB in both groups, with no significant difference between them noted. NMG presented a decrease of 11.9 dB in the air-bone gap, which was a slightly better result than the TFG group, with a decrease of 11.1 dB. This result perhaps demonstrates similar physical characteristics between both grafts when closing previous membrane perforations and establishing integrity of the chain of sound transmission.

Previous studies have shown variable audiological gain results. There is no consensus about the definition of an audiological success. For some authors, air-bone gap closures ranging from 5 dB are already considered success, although for others only gap closures superior to 15 dB can be considered as successful.^27^, ^28^ Stronger scientific evidence studies usually demonstrate an audiological gain varying between 10.8 and 12.5 dB, similar to that shown in the present study.^7^, ^29^

Cases of infection or other major complications were not observed, similar to results in the literature.

Unlike fascia, nasal mucosa is histologically similar to middle ear mucosa. The nasal epithelium produces IgA and presents immunological characteristics that facilitate its adjustment to a highly contaminated environment. Despite concerns surrounding the use of contaminated tissue as a graft, we did not see an increase in the incidence of infection, such as was observed in the study of Strasser and Schratzenstaller.^19^ Moreover, the inferior turbinate used as graft donor area has regeneration potential and may be used for those in a need of a second procedure. Finally, there is no need for external incisions or sutures to harvest the NMG. Nevertheless, the patient may present nasal crust formation along the wound surface, nasal congestion, and small volume nasal bleeding.

## Conclusion

NMG is a safe and effective alternative to be used as a graft in type I tympanoplasty, presenting similar surgical and audiological results when compared to TFG.

## Limitations

Limitations of the study included the lack of a double-blind study design since the graft’s physical characteristics would make it distinguishable during surgery. Also, patients had a short followup period of only six months.

More randomized controlled trials with longer followup times are needed to corroborate the results found in this study.

## Conflicts of interest

The authors declare no conflicts of interest.

## References

[1] Berthold E. (1878). Uebermyringoplastik. Wien Med Bull..

[2] Wullstein H. (1971). The restoration of the function of the middle ear in chronic otitis media. Ann Otol Rhinol Laryngol..

[3] Shea J.J. (1960). Vein graft closure of eardrum perforations. J Laryngol Otol..

[4] Storrs L.A. (1961). Myringoplasty with the use of fascia grafts. Arch Otolaryngol..

[5] Patterson M.E., Lockwood R.W., Sheehy J.L. (1967). Temporalis fascia in tympanic membrane grafting. Arch Otolaryngol..

[6] Perkins R., Bui H.T. (1996). Tympanic membrane reconstruction using formaldehyde-formed autogenous temporalis fascia: twenty years’ experience. Otolaryngol Head Neck Surg..

[7] Indorewala S., Adedeji T.O., Indorewala A., Nemade G. (2015). Tympanoplasty outcomes: a review of 789 cases. Iran J Otorhinolaryngol..

[8] Halik J.J., Smyth G.D. (1988). Long-term results of tympanic membrane repair. Otolaryngol Head Neck Surg..

[9] Silveira F.C.A., Pinto F.C.M., Caldas Neto S.S. (2016). Tratamento do tímpano perfurado com enxerto de celulose bacteriana: ensaio clínico randomizado. Braz J Otorhinolaryngol..

[10] Tan H.E., Santa Maria P.L., Eikelboom R.H., Anandacoomaraswamy K.S., Atlas M.D. (2016). Type I tympanoplasty meta-analysis: a single variable analysis. Otol Neurotol..

[11] Iacovou E., Vlastarakos P.V., Papacharalampous G., Kyrodimos E., Nikolopoulos T.P. (2013). Is cartilage better than temporalis muscle fascia in type I tympanoplasty? Implications for current surgical practice. Eur Arch Oto-Rhino-Laryngol..

[12] Jalali M.M., Motasaddi M., Kouhi A., Dabiri S., Soleimani R. (2016). Comparison of cartilage with temporalis fascia tympanoplasty: a meta-analysis of comparative studies. Laryngoscope..

[13] Jeffery C.C., Shillington C., Andrews C., Ho A. (2017). The palisade cartilage tympanoplasty technique: a systematic review and meta-analysis. Indian J Otolaryngol Head Neck Surg..

[14] Mohamad S.H., Khan I., Hussain S.S. (2012). Is cartilage tympanoplasty more effective than fascia tympanoplasty? A systematic review. Otol Neurotol..

[15] Yang T., Wu X., Peng X., Zhang Y., Xie S., Sun H. (2016). Comparison of cartilage graft and fascia in type 1 tympanoplasty: systematic review and meta-analysis. Acta Otolaryngol..

[16] Hama T., Yamamoto K., Yaguchi Y., Murakami D., Sasaki H., Yamato M. (2015). Autologous human nasal epithelial cell sheet using temperature-responsive culture insert for transplantation after middle ear surgery. J Tissue Eng Regen Med..

[17] Yamamoto K., Yamato M., Morino T., Sugiyama H., Takagi R., Yaguchi Y. (2017). Middle ear mucosal regeneration by tissue-engineered cell sheet transplantation. NPJ Regen Med..

[18] Yamamoto K., Hama T., Yamato M., Uchimizu H., Sugiyama H., Takagi R. (2015). The effect of transplantation of nasal mucosal epithelial cell sheets after middle ear surgery in a rabbit model. Biomaterials..

[19] Strasser G., Schratzenstaller B. (2008). Myringoplasty with autologous mucosa-graft. Laryngorhinootologie..

[20] Dabholkar J.P., Vora K., Sikdar A. (2007). Comparative study of underlay tympanoplasty with temporalis fascia and tragal perichondrium. Indian J Otolaryngol Head Neck Surg..

[21] Sapçi T., Almaç S., Usta C., Karavuş A., Mercangöz E., Evcimik M.F. (2006). Comparison between tympanoplasties with cartilage-perichondrium composite graft and temporal fascia graft in terms of hearing levels and healing. Kulak Burun Bogaz Ihtis Derg..

[22] Solmaz M.A., Yücel E.A., Ozdemir M., Güldiken Y., Değer K. (2002). Comparison of hearing levels and tympanic membrane healing obtained by cartilage palisade and temporal fascia tympanoplasty techniques: preliminary results. Kulak Burun Bogaz Ihtis Derg..

[23] Alkan S., Baylanĉiĉek S., Sözen E., Baŝak T., Dadaŝ B. (2009). Effect of the use of dry (rigid) or wet (soft) temporal fascia graft on tympanoplasty. Indian J Otolaryngol Head Neck Surg..

[24] Glasscock M.E. (1973). Tympanic membrane grafting with fascia: overlay vs. undersurface technique. Laryngoscope..

[25] Gersdorff M., Gérard J.M., Thill M.P. (2003). Overlay versus underlay tympanoplasty: comparative study of 122 cases. Rev Laryngol Otol Rhinol (Bord)..

[26] Lyons S.A., Su T., Vissers L.E., Peters J.P., Smit A.L., Grolman W. (2016). Fascia compared to one-piece composite cartilage-perichondrium grafting for tympanoplasty. Laryngoscope..

[27] Hagemann M., Häusler R. (1992). Tympanoplasty with adipose tissue. Laryngorhinootologie..

[28] Podoshin L., Fradis M., Malatskey S., Ben-David J. (1996). Tympanoplasty in adults: a five-year survey. Ear Nose Throat J..

[29] Haisch A., Harder J., Hopfenmüller W., Sedlmaier B. (2013). Functional and audiological results of tympanoplasty type I using pure perichondrial grafts. HNO..

